# Thermal Mending of Electroactive Carbon/Epoxy Laminates Using a Porous Poly(ε-caprolactone) Electrospun Mesh

**DOI:** 10.3390/polym13162723

**Published:** 2021-08-14

**Authors:** Roberto Cescato, Daniele Rigotti, Haroon Mahmood, Andrea Dorigato, Alessandro Pegoretti

**Affiliations:** 1 Department of Industrial Engineering, University of Trento, Via Sommarive 9, 38123 Trento, Italy; robertocescato@gmail.com (R.C.); haroon.mahmood@unitn.it (H.M.); andrea.dorigato@unitn.it (A.D.); 2 National Interuniversity Consortium of Materials Science and Technology (INSTM), Via Giusti 9, 50121 Florence, Italy

**Keywords:** carbon fibers composites, poly(caprolactone), self-healing

## Abstract

For the first time, a porous mesh of poly(ε-caprolactone) (PCL) was electrospun directly onto carbon fiber (CF) plies and used to develop novel structural epoxy (EP) composites with electro-activated self-healing properties. Three samples, i.e., the neat EP/CF composite and two laminates containing a limited amount of PCL (i.e., 5 wt.% and 10 wt.%), were prepared and characterized from a microstructural and thermo-mechanical point of view. The introduction of the PCL mesh led to a reduction in the flexural stress at break (by 17%), of the interlaminar shear strength (by 15%), and of the interlaminar shear strength (by 39%). The interlaminar fracture toughness of the prepared laminates was evaluated under mode I, and broken samples were thermally mended at 80 °C (i.e., above the melting temperature of PCL) by resistive heating generated by a current flow within the samples through Joule’s effect. It was demonstrated that, thanks to the presence of the electrospun PCL mesh, the laminate with a PCL of 10 wt.% showed healing efficiency values up to 31%.

## 1. Introduction

Polymer composites emerged in the middle of the twentieth century as a promising class of engineering materials, providing new perspectives for structural applications [1]. The ease of manufacturing, design flexibility, lightweight, high strength, low maintenance, elevated durability, lack of corrosion, and multifunctionality are examples of additional assets that can be attributed to this class of materials [2,3]. Despite all these advantages, the monitoring and prediction of composites life span still represents a major problem [4]. The typical failure mechanism of fiber-reinforced polymers (FRPs) refers to interfacial delamination and/or matrix cracking [5,6]. The problem with the repairing of FRPs is that most of the mending methods are time-consuming and/or require a lot of manual interventions. Moreover, the microcracks growing in the bulk of the composite structures are difficult to be detected and repaired. For this reason, the interest towards multifunctional FRPs with self-healing capability has increased in the last years [7].

Generally speaking, the potential advantages of investing in self-healing materials are the minimization of the repairing costs, the extension of the service life of the components, and the elimination of the detrimental effects caused by their uncontrolled degradation. Four concepts must be considered to improve the self-healing efficiency of FRPs: localization, time, mobility, and the repairing mechanism [8]. The concept of localization indicates the position of the damage within the material, while the scale refers to the dimension of the damage (i.e., surface scratches, microcracks, cuts, fiber debonding, or delamination). The factor related to temporality is given by the time gap between the damage event, its repair, and the time needed to heal the material should be the lowest possible. Lastly, the analysis of the repairing mechanism is fundamental, and increasing the mobility of the healing agent inside the damaged area is a key factor to decrease the time necessary to repair a component. Self-healing materials are generally divided into extrinsic and intrinsic healing agents. In the intrinsic (also called non-autonomous) systems, the self-healing properties may not be activated unless they are not triggered externally, while in extrinsic (also called autonomous) systems, the self-healing feature is activated independently [7].

In extrinsic self-healing composites, an external healing agent is generally confined within capsules (or in a vascular system) dispersed within the polymer matrix (in most cases, a thermosetting epoxy resin). When an advancing crack breaks the capsules, the contained healing agent is released and activated by a catalyst (dispersed in the polymer matrix) which promotes its polymerization and consequent sealing of the damage [9]. Generally, the healing agent inside the capsules is in liquid form to promote its mobility when necessary. The flow of the healing agent in the cracked area and the initiation of the repairing action begins without the requirement of external stimuli [10].

In the intrinsic mechanism, the incorporation of self-healing features is directly related to the chemistry of the polymer used as healing agent, without the presence of a catalyst, and the reformation of chemical bonds in the material must be triggered by an external stimulus such as heat, light, or oxygen [10]. The main advantage of this mechanism over the extrinsic one relies upon the theoretically infinite possible repairing cycles, provided that the healing agent is not degraded during the process [11]. From a mechanical and theoretical point of view, the complete healing of an interface can only be realized if the new interface has exactly the same features of the bulk material. This means that the interfaces created by the damage event virtually vanish when the healing mechanism takes place, thanks to the development of chain entanglements and/or physico-chemical crosslinks as strong as the polymer matrix [12]. One of the methods reported to add the healing agent into the composite is by dispersing it as a powder in the epoxy resin [13,14,15,16,17]. This method has been demonstrated to be effective, but the powders tend to form agglomerates, and the sample preparation results tend to be quite complex and time-consuming, as well as the elevated viscosity of the uncured matrix during the processing. In a previous work on self-healing composites of this group, an impermeable film of healing agent constituted by a cyclic olefin copolymer (COC) was inserted in the interlaminar region, but the resulting laminates showed a poor adhesion due to the weak fiber/matrix interface [18].

In order to maintain the pristine viscosity of the uncured resin and a good adhesion between fibers and matrix in the present work, an electrospun polymeric mesh was directly deposited on carbon fiber (CF) plies and used as healing agent. Electrospinning technique can be defined as a process of drawing polymeric fibers from either a polymer solution or polymer melt. Based on an electro-hydrodynamics phenomenon, electrostatic force is used to draw the polymer solution into a liquid-jet form to fabricate non-woven fabrics [19]. The method, however, is affected by several parameters, such as viscosity, operating voltage, temperature, pressure, and flow rate of the solution. Electrospinning is a versatile technique for the preparation of micro or nanofibers. Yao et al. [20] produced an electrospun mesh of PCL to improve the self-healing properties of an epoxy/PCL composite, while Wu et al. [21] produced hybrid self-healing core-shell nanofibers made by dicyclopentadiene, enwrapped into polyacrylonitrile. The resulting composites were subjected to an in-depth microstructural and thermo-mechanical characterization, and the thermal mending capability of PCL was evaluated by comparing the fracture toughness of virgin and healed laminates.

On the basis of these considerations, this research aims to develop, for the first time, carbon fiber reinforced composites in which the self-healing action can be obtained through the presence of an electrospun PCL mesh directly deposited on CF plies. This polymer was selected because of its proven healing efficacy in epoxy matrices [22,23,24]. The insertion of a porous mesh of PCL could maintain the workability of the epoxy resin and a good level of interlaminar adhesion, fundamental features to retain the mechanical properties of the pristine laminates [25]. An in-depth microstructural and thermo-mechanical characterization of the obtained composites was carried out, and the repair was performed by an electro-activated Joule heating effect [26]. The efficiency of the healing action was determined by comparing the interlaminar fracture toughness (G_IC_) of virgin and repaired materials.

## 2. Materials and Methods

### 2.1. Materials

A bi-component epoxy system, provided by Elantas Europe S.r.l. (Collecchio, Italy), was used as thermosetting matrix. It was composed by an epoxy resin (Elantech EC 157.1) and an aminic hardener (Elantech W342), mixed at a ratio of 100:30. Carbon fiber (CF) unidirectional fabrics (GV-201 U TFX), provided by Angeloni s.r.l. (Venice, Italy), were used as reinforcement. It was composed of high strength CF plies (surface density of 200 g/m^2^) and thermoplastic-coated glass yarns (weft, density of 17 g/m^2^). The polycaprolactone (PCL) used in this work was in the form of filaments (PCL99 Naturel), provided by 3D4makers B.V. (Haarlem, The Netherlands) with a diameter of 1.75 mm. According to the datasheet, it had a density of 1.145 g/cm^3^ and a molecular weight (*M_w_*) of 84,500 g/mol.

### 2.2. Samples Preparation

#### 2.2.1. Electrospinning of PCL Fibers

**Figure 1:**
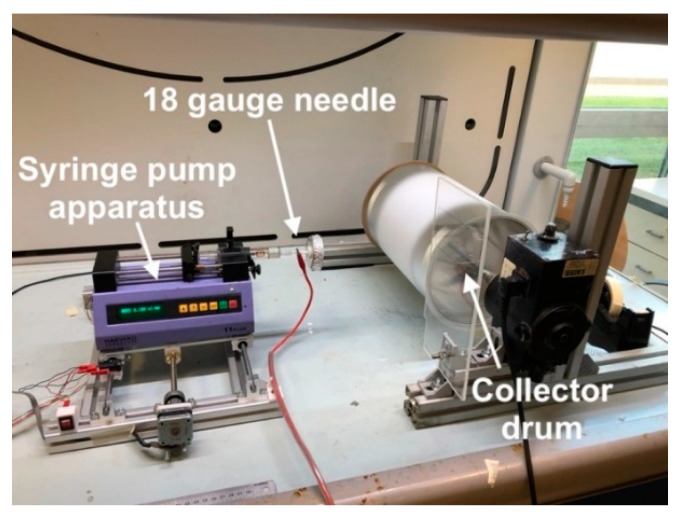
Representative image of the lab made electrospinning apparatus.

The coating of CF fabrics with PCL was performed through an electrospinning technique with a lab-made apparatus shown in Figure 1. Polycaprolactone was dissolved into a solution of dimethylformamide (DMF) and tetrahydrofuran (THF), with a DMF:THF ratio of 20:80 and 30:70 by weight (see Table 1) [27,28]. The solution was stirred for 4 h at room temperature until complete dissolution, and it was then mildly ultrasonicated for 10 min in a Labsonic LBS1 bath (Falc Instruments Srl, Bergamo, Italy), in order to remove small air bubbles formed during the stirring process, and then transferred in a 10 mL glass syringe. The syringe containing the polymer solution was fixed on a Harvard apparatus Model 11 (Harvard apparatus Inc., Holliston, MA, USA), equipped with an 18 gauge needle. The syringe pump apparatus was mounted on an Arduino controlled slider that moved back and forward at 1 m/min alongside the axis of the collector cylinder. A collector drum having a diameter of 20 cm rotated at 300 rpm at a distance of 10 cm from the needle tip. The needle was connected to the positive electrode of a high voltage generator Spellman SL30 (Spellman, Hauppauge, NY, USA) while the drum collector was connected to the ground.

In order to identify the best parameters to obtain a regular electrospun mesh, a 2^4^ full factorial design has been used [29]. Four processing variables (i.e., polymer concentration, DMF concentration in the solution, applied voltage, and flow rate) were varied from a lower (−1) to an upper (+1) value, while the diameter of the electrospun fibers was taken as yield (y). These variables and their respective levels are resumed in Table 1. These values were chosen according to previous literature works [27,30,31].

Analysis of variance (ANOVA) technique was used to evaluate the significance of the input parameters and their interaction effects on the measured responses, according to a linear model expressed by Equation (1):

(1) $$y=\overset{k}{\underset{i=1}{\sum }}\beta_{i}x_{i}+\overset{k-1}{\underset{i=1}{\sum }}\overset{k}{\underset{j=i+1}{\sum }}\beta_{ij}x_{i}x_{j}+\epsilon$$

where *y* is the measured output, *x_i_* is the designated input variable, *β_ij_* is the regressor coefficient and *ε* is the error term. The terms with substantial *F* value (Fisher test), and thus low probability value (*p* < 0.05), were selected as the statistically significant parameters [29].

SEM images of the electrospun meshes were taken with a Supra 40 (Carl Zeiss AG, Oberkochen, Germany) microscope after Pt-Pd sputtering, and the diameter of the obtained fibers was obtained from SEM micrographs by using an ImageJ 1.51, applying several algorithms upon image segmentation [32]. After the optimization of the electrospinning parameters, the deposition on CFs was performed for a certain time, so that the nominal final concentrations of PCL in the composite laminate was 5 wt.% and 10 wt.%. The time needed to obtain these concentrations was determined knowing the area of deposition (equal to 1260 cm^2^), the rate of deposition (0.1 mL/min) and the concentration of the PCL solution (0.2 g/mL). Therefore, deposition times equal to 150 and 300 min were selected. The resulting CF fabrics coated with PCL were cut in square plies having a width of 15 cm and length of 20 cm, in order to prepare composite laminates.

#### 2.2.2. Preparation of the Composites

Composites were prepared with a hybrid technique, combining hand lay-up and vacuum-assisted resin transfer molding (VARTM), in order to avoid incomplete permeation of the resin within the laminates and to minimize the concentration of voids. Hand lay-up technique involved manually placing and impregnating each fiber fabric with resin using a brush. The impregnated laminate was inserted into the VARTM system and resin infusion was performed under vacuum until excess of resin was flown out of the laminate. To avoid the melting of the electrospun PCL mesh, an optimized curing cycle of 8 h at room temperature and 40 h at 50 °C was performed under a pressure equal to 0.8 MPa, by using a Carver 2699 hot press (Carver Inc., Wabash, IN, USA). In this way, four-layered and sixteen-layered laminates of neat epoxy/CF laminates and composites having a PCL concentration of 5 wt.% and 10 wt.% were prepared. Four-layered laminates were utilized for flexural tests and electrical resistivity measurements, while sixteen-layered composites were considered in short beam shear strength and interlaminar fracture toughness tests. Specimens for interlaminar fracture toughness tests were prepared inserting a polyethylene terephthalate (PET) film 26 μm thick on one side in the middle section of each sample, following ASTM D5528 standard [33]. This operation was performed to create a pre-crack, as required by interlaminar fracture toughness tests. The designation and the nominal composition of the prepared laminates is reported in Table 2. 

### 2.3. Experimental Techniques

#### 2.3.1. Characterization of the Laminates

SEM analysis was performed to measure the diameter of electrospun PCL fibers on carbon fibers, to evaluate the thickness of the electrospun layer deposited on CF plies and to analyze the morphology of PCL domains on the crack propagation surfaces of virgin and healed samples. All the specimens were metalized with a platinum/palladium alloy (80:20) coating for 20 s and observed with a Supra 40 microscope (Carl Zeiss AG, Oberkochen, Germany). The microstructure of the composites was analyzed by using a Axiophot optical microscope (Carl Zeiss AG, Oberkochen, Germany), coupled with a Leica DC300 digital camera (Leica Microsystems Ltd., Heerbrugg, Switzerland). The samples were put in a removable plastic mold and then incorporated in epoxy resin and cured for 24 h at room temperature. Then, the samples were removed from the molds and the surface was polished by abrasive grinding paper made of silicon carbide with grit polishing sizes of 240, 800, 1200, and 4000, sequentially, and then polished with 3 μm and 1 μm cloths. The polishing system LaboPol-5 (Struers, Copenhagen, Denmark) was set at a speed of 200 rpm.

The density of each sample was measured at 23 °C by using a ME104 (Metter-Toledo, Columbus, OH, USA) precision balance, having a sensitivity of 10^−4^ g. To calculate the volume fraction of voids, it was necessary to measure the density of the PCL mesh and carbon fibers. This was done through a AccuPyc 1330 (Micromeritics, Norcross, GA, USA) helium pycnometer, equipped with a chamber of 1 cm^3^, performing 30 runs for each measurement. The composites were weighed in air and in ethanol following ASTM D792 standard [34], and the experimental density ($\rho_{exp}$) was calculated with Equation (2):

(2) $$\rho_{exp}=\frac{w_{air}}{w_{air}-w_{eth}}\;\left(\rho_{eth}-\rho_{air}\right)+\rho_{air}$$

where $w_{air}$ and $w_{eth}$ are the weight of the samples in air and ethanol, respectively, while $\rho_{air}$ and $\rho_{eth}$ are the density, measured in air and ethanol, respectively. The theoretical density of the composites ($\rho_{t}$) was evaluated with Equation (3):

(3) $$\rho_{t}=\frac{1}{\frac{w_{CF}}{\rho_{CF}}+\frac{w_{M}}{\rho_{M}}+\frac{w_{PCL}}{\rho_{PCL}}}$$

where $w_{CF}$, $w_{M}$, and $w_{PCL}$ are the weight fractions of carbon fibers, matrix (epoxy + hardener) and PCL, respectively, while $\rho_{CF}$, $\rho_{M}$ and $\rho_{PCL}$ are the density of the constituents. For all the samples $\rho_{CF}$ was equal to 1.78 g/cm^3^ and $\rho_{M}$ to 1.15 g/cm^3^ while $\rho_{PCL}$ was measured after 150 min of deposition resulting 1.0438 ± 0.0428 g/cm^3^, and 1.0956 ± 0.0244 g/cm^3^ after 300 min. Finally, the volume fraction of voids ($V_{v}$) was calculated as reported in Equation (4):

(4) $$V_{v}=\frac{\rho_{t}-\rho_{exp}}{\rho_{t}}$$

The evaluation of the weight fraction of the constituents was utilized to confirm the calculation of the amount of PCL inside the laminates.

Thermogravimetric analysis was performed to obtain information about the thermal degradation behavior of the samples. These tests were carried under a nitrogen flow of 100 mL/min, by using a TG50 MT5 (Metter-Toledo, Columbus, OH, USA) thermobalance, setting a heating rate of 10 °C/min from 30 °C to 700 °C. This test allowed the calculation of $T_{1\%}$ and $T_{5\%}$, i.e., the temperature corresponding to a mass loss of 1% and 5%, of $T_{d}$, i.e., the peak temperature of the mass loss derivative for EP and PCL phases, and of $m_{700}$, i.e., the residual mass at 700 °C.

To evaluate the flexural properties of unhealed samples, a three-point flexural test was performed by using an Instron 5969 (Instron, Norwood, MA, USA) universal testing machine, equipped with a 50 kN load cell, following ASTM D790 standard. Rectangular specimens, having a width of about 13 mm and a thickness about 1 mm, were cut from four layered laminates [35]. According to the standard, a span to depth ratio of 60:1 was imposed for the flexural modulus measurements, while a ratio of 40:1 was fixed for the determination of the flexural strength. A crosshead speed corresponding to a strain rate on the outer surface of the samples equal to 0.01 mm^−1^ was utilized. At least five specimens were tested for each composition. Flexural stress and flexural strain were calculated according to the Equations (5) and (6).

(5) σf=3PL2bd2

where: *σ* = stress in the outer fibers at midpoint, MPa; *P* = load at a given point on the load-deflection curve, N; *L* = support span, mm; *b* = width of beam tested, mm; *d* = depth of beam tested, mm.

(6) εf=6DdL2

where: *ε_f_* = strain in the outer surface, mm/mm; *D* = maximum deflection of the center of the beam, mm; *L* = support span, mm; *d* = depth, mm.

Evaluation of interlaminar shear strength (ILSS) of the prepared composites was performed through short beam shear strength tests, following ASTM D2344 standard [36]. An Instron 5969 (Instron, Norwood, MA, USA) universal testing machine, equipped with a 50 kN load cell, was utilized imposing a crosshead speed of 1 mm/min. Specimens were cut in a way that their length and width were 6 and 2 times their thickness, respectively. At least five specimens were tested for each composition. The ILSS was calculated according to Equation (7):

(7) $$ILSS=0.75\frac{P_{max}}{b\;h}$$

where $P_{max}$ is the maximum load, *b* and *h* are the width and thickness of the specimens, respectively.

The fracture behavior of the laminates was evaluated through mode I interlaminar fracture toughness tests, according to ASTM D5528 standard [33]. An Instron^®^ 5969 universal testing machine, equipped with a 50 kN load cell, was utilized to test double cantilever beam (DCB) specimens, 150 mm long and 23 mm wide, cut from 16 layered samples.

**Figure 2:**
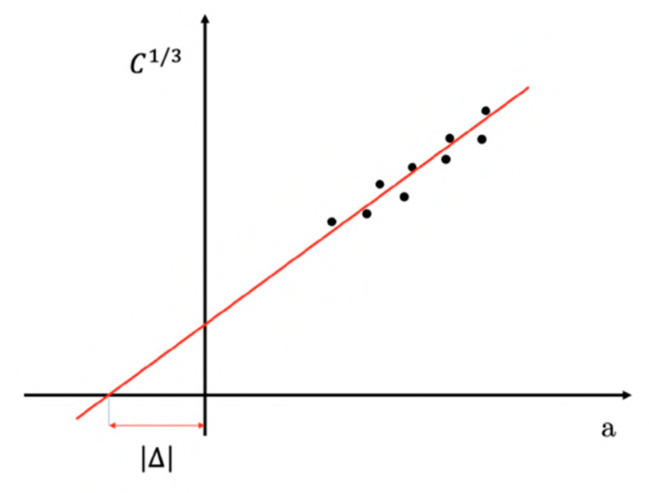
Example of determination of Δ (corrective factor) according to ASTM D5528.

The tests were performed imposing a crosshead speed of 2.5 mm/min, and at least five specimens were tested for each composition. Two loading blocks were bonded to the specimen 50 mm far from the starting crack tip. In order to monitor the crack advancement during the test, a digital webcam B910HD (Logitech, Lausanne, Switzerland) was utilized. To measure the crack propagation, samples were colored in white and a graduated scale of 70 mm with 2 mm accuracy was drawn on the lateral side of the specimens. In this way, it was possible to estimate the crack length (*a*) at each value of stress and strain. The mode I interlaminar fracture toughness (*G_I_*) was then calculated by using the Equation (8):

(8) $$G_{I}=\frac{3\;P\;\delta }{2\;b\;\left(a+\left|∆\right|\right)}$$

where *P* is the applied load, *δ* is the crack opening displacement, *b* is the thickness of the sample and Δ is a corrective factor. According to the ASTM D5528 standard, this corrective factor may be determined experimentally by generating a least squares plot of the cube root of compliance (C^1/3^) as a function of delamination length (*a*) (Figure 2).

The interlaminar fracture toughness of both virgin and healed samples was evaluated considering the point of deviation from linearity (NL), where the load-displacement curve starts to deviate from linearity. In this way, it was possible to evaluate the interlaminar fracture toughness of virgin samples ($G_{Iv}$). Broken specimens were then healed according to the procedure reported in Paragraph 2.3.2 and tested again, to obtain the interlaminar fracture toughness of healed samples ($G_{Ih}$). The healing efficiency ($\eta_{G_{I}}$) was evaluated using the expression reported in Equation (9):

(9) $$\eta_{G_{I}}=\frac{G_{Ih}}{G_{Iv}}\cdot 100$$

#### 2.3.2. Healing Process of the Composites

The effectiveness of the healing through Joule heating effect is strongly dependent on the resistivity of samples. For this reason, resistivity measurements were performed on the prepared composites by adopting a four-probe configuration. Rectangular specimens having a length 40 mm and a width 13 mm were cut from four layered laminates, and the lateral side of each sample was covered with silver paint, to improve the conductivity of the surfaces in contact with the testing electrodes [37,38].The experimental setup was composed by a DC electricity generator IPS 303DD (ISO-TECH Kunststoff GmbH, Ahaus, Germany) and two digital multimeters IDM 67 (ISO-TECH Kunststoff GmbH, Ahaus, Germany). The current and voltage passing through the samples were recorded at selected output voltages equal to 0.1, 0.2, 0.3, 0.4, and 0.5 V. At least five specimens were tested for each composition. An important parameter to consider in these measurements was the pressure applied on the specimens. In fact, the electrical resistivity is influenced by the pressure: compression stresses in the through-thickness direction produce a decrease in the electrical resistivity. On the other hand, a compression stress in the fiber direction produces an increase in the electrical resistivity [39,40]. A compressive force to the mobile electrode was applied by means of an apparatus that transformed an applied torque into a translational force. This was done both to hold the sample during the test and to ensure that the same compression force was always applied, setting the minimum applicable tightening torque of 0.1 cNm. After the records of voltage and the current passing through the samples, the four-point resistivity (ρ) was evaluated using the Equation (10):

(10) $$\rho =\frac{V\;w\;h}{I\;l\prime }$$

where *V* is the voltage, *w* is the width of the sample, *h* is the thickness of the sample, *I* is the current flowing through the sample, and *l′* is the distance between the contact points of the voltmeter.

**Figure 3:**
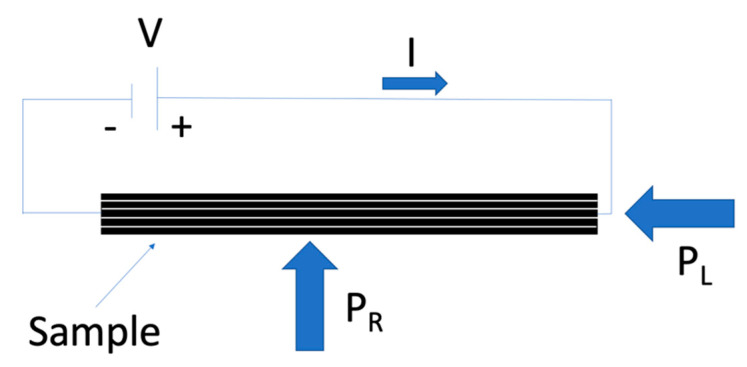
Schematic drawing of the Joule heating repairing device.

As reported in the introductive section, the Joule heating mechanism was adopted to promote the self-healing of the laminates. The lab-made self-healing device was composed of two steel electrodes covered with copper plates, to enhance their electrical conductivity, while the current was applied through electrical terminals. A layer of PET was inserted between each copper plate and steel, to decrease the electrical losses through the steel supports converging the current inside the tested specimen. One electrode was fixed, while the other one was free to move in order to close the sample between them. The pressure applied to the lateral surface of the sample (*P_L_*) was 180 kPa, calculated knowing that the applied torque was equal to 0.1 cNm. According to a previous paper of our group [18], the repairing pressure (*P_R_*) applied to close the crack was set equal to 500 kPa. A piece of glass-ceramic was inserted between each side of the vice’s shoulders and the specimen to minimize heat losses through the steel vice. The specimen was separated from the glass-ceramic through a polytetrafluoroethylene (PTFE) sheet, to ease the detachment of the sample. Before inserting the sample in the lab-made device, as in the electrical resistivity measurements, the lateral surfaces of specimens were covered with silver paint to increase the electrical conductivity [38]. A schematic drawing of the repairing device is shown in Figure 3.

**Figure 4:**
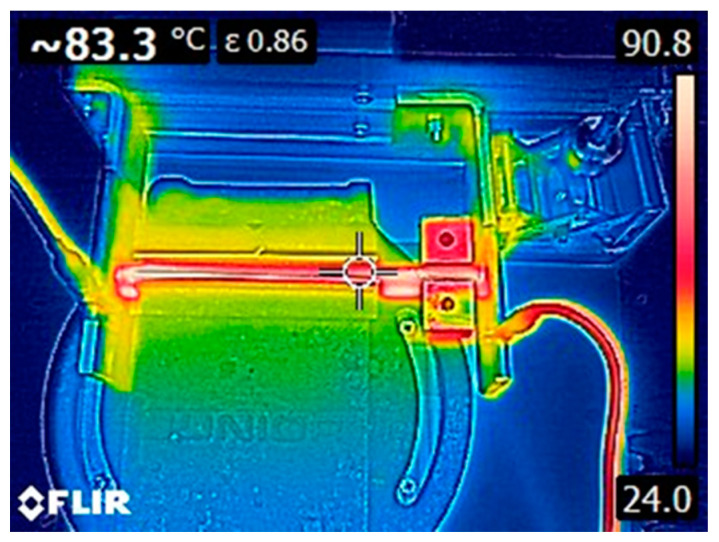
Top view obtained with thermal camera of the EP-CF-b sample during the repairing process.

A 6674A DC (Agilent Technologies, Inc., Santa Clara, CA, USA) generator was used to apply voltage and current to the specimens. The voltage and the current were manually set to obtain a temperature around 80 °C on the crack surface, kept for 30 min, as suggested in literature for similar systems [22]. During the healing process, the temperature was monitored by using an infrared thermal camera E6 (FLIR Systems S.r.l., Limbiate, Italy). A representative image showing the temperature profile captured with the thermal camera during the healing of the EP-10PCL-CF-b sample is represented in Figure 4. It can be seen that during the healing process the sample presents higher temperature near the contact point with the electrodes (90 °C), while on the central zone the temperature is slightly higher than 80 °C. A variability lower than 10 °C was considered acceptable, also considering the difficulty of maintaining a homogeneous temperature profile during the healing process.

## 3. Results and Discussions

### 3.1. Characterization of PCL Electrospun Meshes

**Figure 5:**
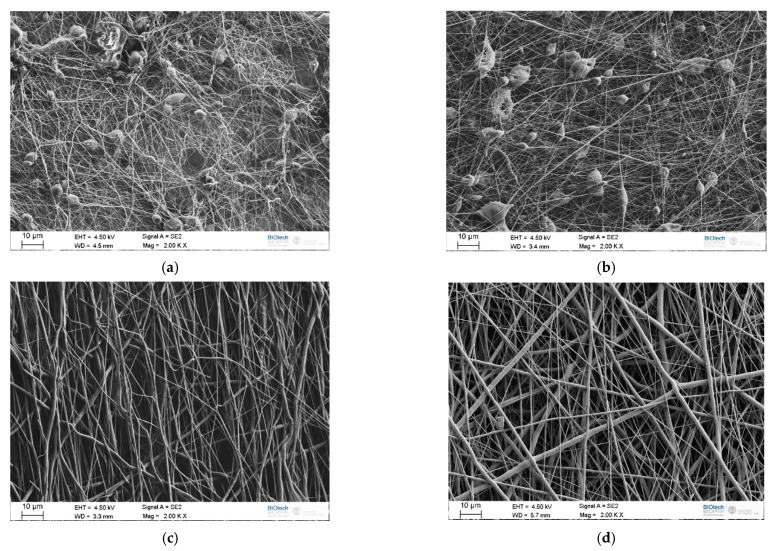
SEM micrographs of some representative electrospun PCL meshes. (**a**) Trial 1, (**b**) trial 8, (**c**) trial 9 and (**d**) trial 16 (see Table 3 for the definition of the process parameters).

Electrospun PCL webs were produced according to electrospinning parameters reported in Table 3, and in Figure 5a–d some representative SEM micrographs of the obtained meshes are reported.

In Figure 5a,b it is possible to notice the presence of a large number of beads, which is one of the most common defects encountered in the electrospinning process [41]. In particular, these two images are related to samples produced with a lower polymer concentration. Micrographs of meshes prepared with a higher polymer concentration, represented in Figure 5c,d, show the presence of fibers with a uniform morphology and without beads. Comparing the morphology of the fibers for two different solution concentrations (Figure 5a,b vs. Figure 5c,d) it is possible to observe how a tailored polymer concentration in the spinning solution can lead to fibers having a good homogeneity and without beads. Performing the electrospinning process with these working parameters allows the preparation of fibers with stable, predictable, and optimized features [42]. Fibers spun from a solution with a lower polymer concentration are characterized by smaller diameters (0.11–0.12 µm, see Figure 5a,b), while fibers produced with a higher concentration have a mean size in the range 0.35–0.39 µm, as shown in Figure 5c,d.

**Figure 6:**
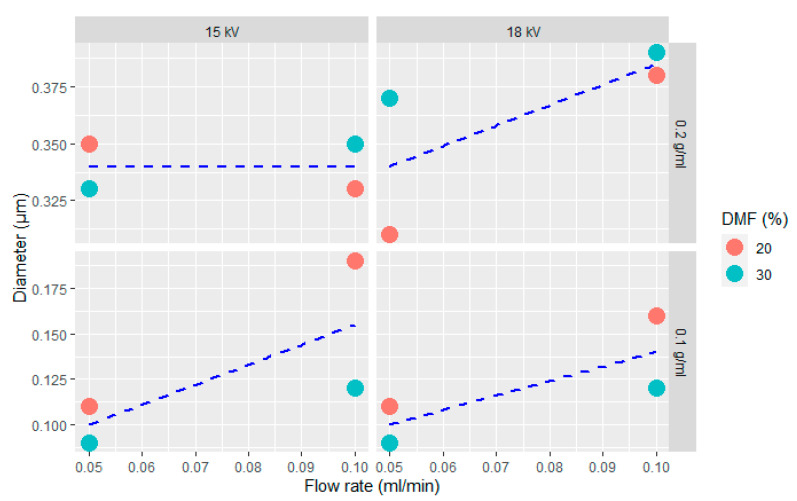
Scatter plots of the diameter of electrospun PCL fibers as a function of the flow rate for different voltage levels (columns), PCL concentration (rows) and DMF content (colors).

Mean diameter of the obtained fibers is summarized in Table 3, while the trend of the fiber size as a function of the process parameters is graphically represented in Figure 6. According to ANOVA, polymer concentration in the solution is the main variable that affects the fiber size, with a *p*-value lower than 10^−5^. Increasing the PCL concentration in the solution leads to an increase in the mean diameter of the fibers and to a widening of the statistical distribution, as already reported in literature [43]. An enhancement of the polymer concentration corresponds to an increase in the viscosity of the solution, hence the electrical charges that initiate the spinning could be insufficient to stretch the polymer solution to a lower fiber diameter. As already seen in Figure 5a,b, a low polymer concentration is also responsible of the formation of beads, due to the fragmentation of the entangled polymer chains before reaching the collector [44]. This effect can be suppressed by increasing the polymer concentration above a certain critical level. In these conditions, chain entanglements overcome the surface tension of the solution and lead to the formation of uniform and bead-less electrospun nanofibers [45].

Fiber diameter is also significantly affected by the flow rate of the solution (*p*-value < 0.05), although to a lesser extent with respect to the polymer concentration. The coupled effect of polymer concentration and DMF content slightly affects the diameter of the fibers (*p*-value ~ 0.05), due to the possible interaction between solution viscosity and its electrical charge density. However, the increased electrical charge density given by the increase in DMF content is hindered at elevated polymer concentration by the increased viscosity. Applied voltage does not substantially affect the diameter of the fibers, at least in the interval from 15 kV to 18 kV. After this preliminary study, the combination of electrospinning parameters selected to coat the CF fabrics is related to trial 16, performed utilizing a polymer concentration of 0.2 g/mL, a DMF amount of 30%, an applied voltage of 18 kV and a flow rate of 0.1 mL/min (see Table 3). In this way, it is possible to obtain a uniform and beads-free web as well as a rather elevated productivity.

Electrospun free-standing PCL meshes, deposited for 150 min and 300 min on CFs according to the conditions of trial 16, were observed through SEM, in order to measure their thickness. The thickness of the mesh after 150 min of deposition was 80 ± 4 μm, while the thickness after 300 min was 162 ± 8 μm, meaning that the thickness of the web is directly proportional to the time of deposition.

### 3.2. Characterization of the Composites

**Figure 7:**
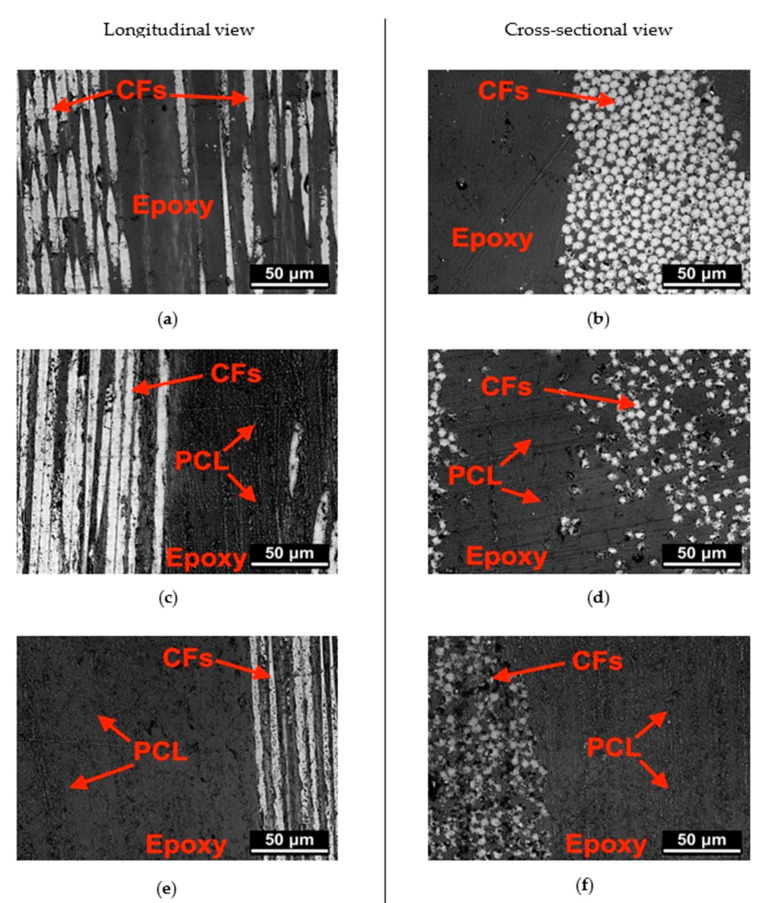
Optical microscope images of unhealed samples (longitudinal and cross-sectional view). (**a**,**b**) EP-CF-b, (**c**,**d**) EP-5PCL-CF-b; (**e**,**f**) EP-10PCL-CF-b samples.

Optical microscopy analysis was conducted on unhealed samples to observe the microstructure of the laminates. In Figure 7a,c,e the optical images of the laminates in the longitudinal view are reported, while Figure 7b,d,f show the micrographs taken in the cross-sectional direction. It can be noticed that in the neat laminate (Figure 7a,b) the epoxy resin presents a smooth surface, while in the other samples the matrix shows a rough surface due to the presence of the electrospun PCL mesh (Figure 7c–f). This confirms the effective diffusion of the epoxy resin throughout the composite laminate, i.e., both within the electrospun mesh and the CF plies.

Increasing the amount of PCL in the composites, the theoretical density tends to decrease, as reported in Table 4. This can be due to the fact that the porous mesh of PCL present in the laminates is not completely impregnated by the epoxy resin. Another reason could be that increasing the thickness of the composite, the pressure of the vacuum pump is not enough to squeeze the layers inside the vacuum bag, leaving air trapped in the laminate during the hand lay-up process. However, the obtained void concentration values can be acceptable for all the samples, and their mechanical properties should not be strongly deteriorated. From Table 4, it can be seen that the nominal amount of PCL deposited in the laminates is very near to the experimental one (*w_PCL_*). Moreover, by increasing the PCL concentration, more epoxy resin is trapped into the mesh, leading to a lower CF content and deteriorating of the mechanical properties.

**Figure 8:**
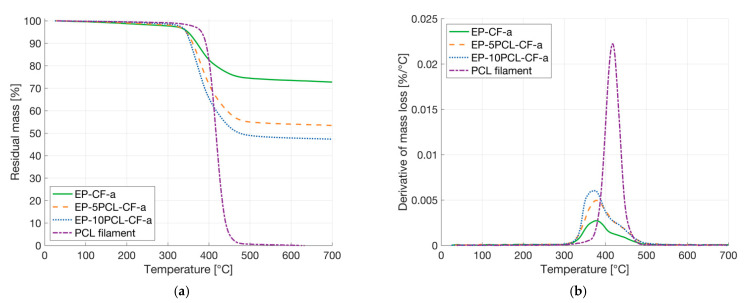
TGA analysis on the prepared laminates and on the PCL filament. (**a**) Residual mass and (**b**) derivative of the mass loss as a function of the temperature.

TGA was performed to obtain information about the thermal degradation resistance of the laminates. The same analysis was performed also on the PCL filament, the thermograms are reported in Figure 8a,b, while the most important results are collected in Table 5. As expected, PCL degrades completely, while the laminates degrade only partially, because of the presence of the reinforcing fibers. Consequently, the residual mass at 700 °C decreases with the PCL amount inside the laminates. As reported in Table 5, a progressive increase in T_1%_ values with the PCL introduction can be detected, suggesting an improved thermal degradation resistance. This is due to the fact that the degradation temperature of the epoxy (378 °C) is lower than that of the PCL matrix (418 °C), and it is not substantially influenced by the PCL addition within the laminates. However, the degradation peaks of the epoxy and PCL phases are partially overlapped, and they cannot be clearly distinguished. This is also because of the limited PCL amount in the composites.

**Figure 9:**
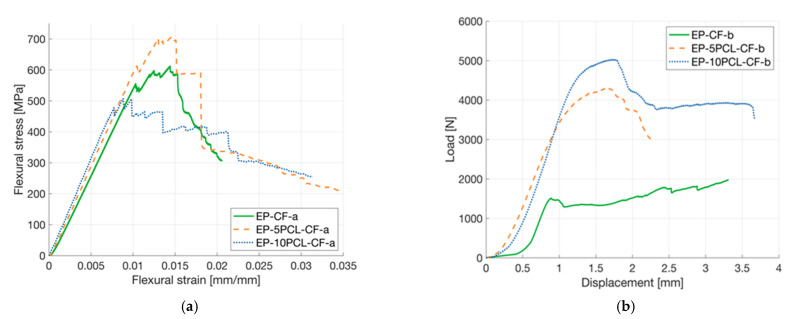
(**a**) Representative stress–strain curves from flexural tests on the prepared composites, (**b**) representative load-displacement curves from short beam strength tests on the prepared laminates.

Flexural tests were conducted on the four-layered laminates, and representative stress-strain curves are represented in Figure 9a. All the samples present a brittle behavior and the insertion of PCL in the laminates does not seem to dramatically influence the mechanical behavior of the composites. The results of flexural tests are summarized in Table 6. The flexural modulus of EP-5PCL-CF-a and EP-10PCL-CF-a samples are slightly higher than that of the neat laminate. However, considering the standard deviation values associated to these measurements, it can be concluded that the prepared laminates have a similar stiffness. A 12% increase in flexural strength is observed for EP-5PCL-CF-a sample compared to a neat EP-CF-a composite, while a limited decrease in strength can be detected for higher PCL amounts (about −17%). The flexural strain at break tends to slightly decrease with the PCL concentration, but even in this case, the observed drop is limited (−15%). It can therefore be concluded that the presence of a porous PCL mesh within the laminates does not significantly affect their flexural properties.

Short beam shear (SBS) test was performed to evaluate the interlaminar adhesion degree in the prepared laminates. Representative load-displacement curves are reported in Figure 9b, while the most important results are summarized in Table 6. Thanks to the higher thickness, the composites with PCL present considerably higher P_m_ values. However, the ILSS value decreases with the PCL amount, and the EP-10PCL-CF-b has an ILSS equal to the 60% of that shown by the EP-CF-b composite. It can therefore be concluded that the insertion of a limited amount of PCL in the laminates leads to a decrease in the interlaminar adhesion, but it is important to underline that the observed drop is not dramatic if compared with that observed in our previous work [18]. In that paper, a decrease in the ILSS of about 70%, with respect to the neat laminate, was observed, introducing the healing agent in the form of thin films. This can also explain why the failure properties of the laminates in flexural conditions are not dramatically affected by the PCL insertion.

### 3.3. Evaluation of the Healing Efficiency

The voltage-current relationship for each sample was investigated by carrying out resistivity measurements. A linear trend was noticed between the voltage and current (not shown for the sake of brevity), keeping the increase in temperature as low as possible by quickly registering the values of voltage and current. In fact, it is well known that, for these materials, the resistivity increases with the temperature. As shown in Table 7, the mean value of the resistivity tends to slightly decrease with the PCL concentration, but, considering the standard deviation values, it can be concluded that the PCL addition does not strongly affect the resistivity of the samples, allowing the application of the Joule heating effect as a repairing mechanism. This result could be expected, since carbon fibers are the main component of the laminates (even if CF content decreases with the PCL concentration), and they have much higher electrical conductivity (~10^5^ S/m) than epoxy resin (~10^−15^ S/m) and PCL (~10^−10^ S/m) [46,47].

**Figure 10:**
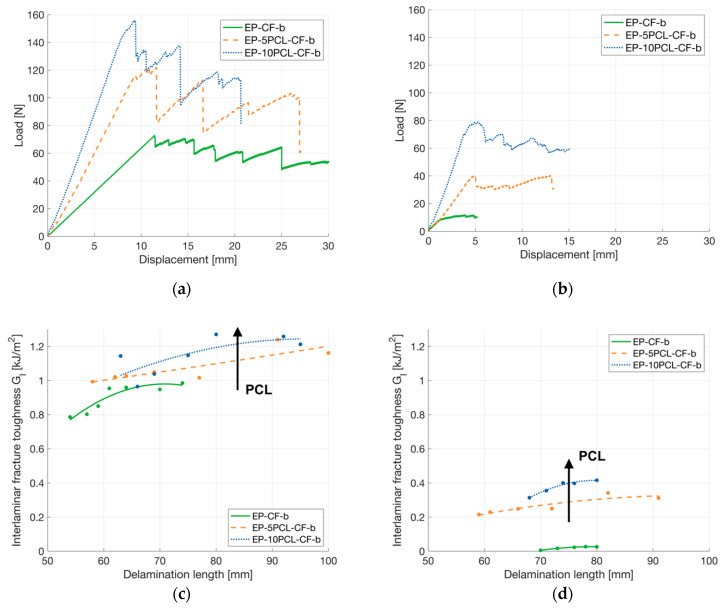
Results of mode I interlaminar fracture toughness tests on the prepared laminates. Representative load-displacement curves of (**a**) virgin and (**b**) healed samples. Representative trend of G_I_ as a function of the delamination length in (**c**) virgin and (**d**) healed samples.

For the evaluation of the fracture behavior of the prepared laminates, interlaminar fracture toughness tests were performed on virgin and healed samples (see Figure 10a,b) while their representative G_I_ values, corresponding to delamination length for each sample, are shown as a delamination resistance curve (R curve) in Figure 10c,d. In Figure 10a, representative load-displacement curves of unhealed laminates are reported. It can be seen that each sample exhibits a brittle behavior, and each load drop corresponds to the propagation of the crack. It can be seen from Table 8 that the G_I_ values of EP-5PCL-CF-b and EP-10PCL-CF-b samples are 25% higher than that of the neat laminate. The increase in G_I_ due to the presence of PCL was also observed in a previous paper on PCL nanofibers within a PA66 matrix [48]. It can be hypothesized that the presence of a relatively soft PCL mesh could lead to a nanofibers bridging effect across the crack-plane, contributing to an increased toughness in the laminates [49].

After the thermal mending process, each specimen was tested again in the same conditions to evaluate the healing efficiency. The fracture behavior is more ductile than that displayed by unhealed samples, as shown in Figure 10b. This could be attributed to the presence of a diffused thermoplastic PCL phase in the crack propagation surface. Looking at the delamination resistance curves (shown in Figure 10c,d), it can be seen that the sample EP-CF-b is not significantly repaired, since the maximum sustained load is 10 N (about 85% lower than that of the virgin specimen). On the contrary, the maximum load sustained by the healed EP-5PCL-CF-b and EP-10PCL-CF-b samples is almost halved with respect to the corresponding virgin composites. This confirms the partial mending of the laminates due to the presence of the PCL phase. The numerical results of these tests in terms of maximum load (*P_MAX_*), G_I_ calculated at NL point (G_I_^NL^) and the healing efficiency ($\rho_{G_{I}}^{NL}$) are reported in Table 8.

**Figure 11:**
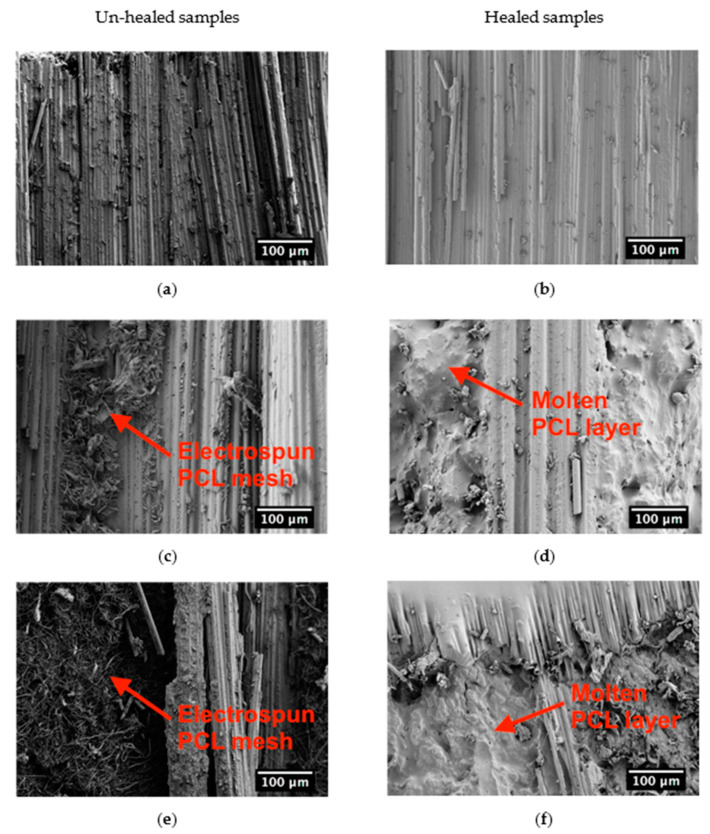
SEM micrographs of the delamination surface of unhealed and healed samples. (**a**,**b**) EP-CF-b, (**c**,**d**) EP-5PCL-CF-b and (**e**,**f**) EP-10PCL-CF-b laminates.

As expected, the healing efficiency in EP-CF-b sample is almost zero. On the other hand, healing efficiency values of EP-5PCL-CF-b and EP-10PCL-CF-b samples are 15% and 32%, respectively. SEM micrographs were taken on the crack propagation surfaces of virgin and healed samples to analyze the morphology of the PCL mesh before and after the mending process. This comparison is reported in Figure 11a–f. As expected, no visual changes can be observed in the EP-CF-b sample after the healing process, as visible in Figure 11a,b. On the contrary, in EP-5PCL-CF-b and EP-10PCL-CF-b virgin samples the PCL mesh is still visible (Figure 11c–e), while in the healed ones the PCL nanofibers web is replaced by a molten layer of PCL (Figure 11d–f). This structure is the result of the PCL melting and flow on the damaged area during the mending process, meaning that the selected repairing time and temperature conditions are suitable to activate the mending of the samples.

**Figure 12:**
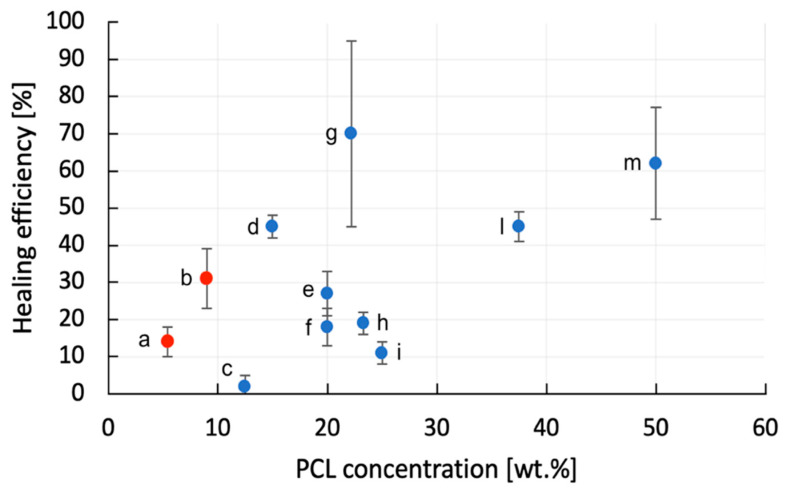
Comparison of the healing efficiency values obtained in the present work and in some papers on similar systems (see Table 9 for the system codes).

In order to evaluate the real extent of the innovation related to the introduction of an electrospun PCL mesh as healing material, it could be important to compare the healing efficiency values obtained in the present work with those reported in literature for similar systems. In Table 9 the details of the papers reported in literature on the self-healing behavior of epoxy/PCL systems (with or without the presence of reinforcing fibers) are summarized, while in Figure 12, the healing efficiency values are represented as a function of the PCL concentration. Even if the PCL concentration utilized in the present study was rather small if compared to that considered in the other papers, the healing efficiency values obtained in this work are considerable, especially with a PCL content of 10 wt.%. The physical structure of an electrospun mesh allows both an optimal flow of the healing agent in the cracks and the diffusion of the epoxy resin inside the mesh. Moreover, the flexural properties of the laminates with the electrospun mesh are comparable with those of the neat laminates, and only a slight decrease in the ILSS can be detected. Electrospinning technique introduces also processability advantages, as the avoidance of the viscosity increase in the uncured resin due to the introduction of thermoplastic healing particles, present in many self-healing composite systems. It can therefore be concluded that the electrospinning technique can be a suitable process to produce meshes able to impart self-healing properties to structural composites.

## 4. Conclusions

In this work, a porous mesh of PCL was produced through electrospinning and directly deposited on unidirectional carbon fiber fabrics, to create a multifunctional laminate with self-healing properties. At this aim, neat epoxy/CF laminates and composites with a nominal PCL concentration of 5 wt.% and 10 wt.% were prepared. The maximum void concentration inside the laminates was about 3%, confirming the successful infusion of the resin within the mesh. The introduction of an electrospun PCL layer tended to slightly decrease the failure properties of the laminates under flexural conditions, but an acceptable level of interlaminar adhesion was maintained even at elevated PCL amounts. An electro-activated mending process was utilized to repair the broken specimens, heating them and applying an electrical voltage. GI values of the laminates were measured before and after the mending process, and a healing efficiency up to 32% was obtained with a PCL amount of 10 wt.%, thanks to the effective diffusion of the molten PCL layer within the fracture zone. The possibility of partially healing composite laminates was therefore demonstrated by introducing a very limited PCL amount through electrospinning, while retaining their processability and without substantially impairing their interlaminar resistance.

## Figures and Tables

**Table 1:** Electrospinning variables and their respective levels.

Code	Variable	Value = −1	Value = +1
x1	polymer concentration (g/mL)	0.1	0.2
x2	DMF concentration (wt.%)	20	30
x3	applied voltage (kV)	15	18
x4	flow rate (mL/min)	0.05	0.1

**Table 2:** List of the prepared laminates.

	CF Layers	PCL Layers	PCL (wt.%)	Thickness (mm)
EP-CF-a	4	0	0	1.2
EP-5PCL-CF-a	4	3	5	1.2
EP-10PCL-CF-a	4	3	10	1.3
EP-CF-b	16	0	0	3.3
EP-5PCL-CF-b	16	15	5	5.3
EP-10PCL-CF-b	16	15	10	7.2

**Table 3:** Experimental trials for the optimization of electrospinning process parameters.

Trial	Polymer Concentration in Solution [g/mL]	DMF Concentration in Solution [%]	Applied Voltage [kV]	Solution Flow Rate [mL/min]	Diameter of the Fibers [μm]	Microstructural Defects
1	0.1	20	15	0.05	0.11 ± 0.04	Beads
2	0.1	30	15	0.05	0.09 ± 0.04	Beads
3	0.1	20	18	0.05	0.11 ± 0.02	Beads
4	0.1	30	18	0.05	0.09 ± 0.04	Beads
5	0.1	20	15	0.10	0.19 ± 0.06	Beads
6	0.1	30	15	0.10	0.12 ± 0.04	Beads
7	0.1	20	18	0.10	0.16 ± 0.06	Beads
8	0.1	30	18	0.10	0.12 ± 0.06	Beads
9	0.2	20	15	0.05	0.35 ± 0.11	-
10	0.2	30	15	0.05	0.33 ± 0.13	-
11	0.2	20	18	0.05	0.31 ± 0.12	-
12	0.2	30	18	0.05	0.37 ± 0.15	-
13	0.2	20	15	0.10	0.33 ± 0.12	-
14	0.2	30	15	0.10	0.35 ± 0.12	-
15	0.2	20	18	0.10	0.38 ± 0.14	-
16	0.2	30	18	0.10	0.39 ± 0.19	-

**Table 4:** Density and relative composition of the prepared laminates.

	*ρ_exp_* [g/cm^3^]	*ρ_t_* [g/cm^3^]	*w_PCL_* [wt.%]	*w_EP_* [wt.%]	*w_CF_* [wt.%]	*V_PCL_* [vol.%]	*V_EP_* [vol.%]	*V_CF_* [vol.%]	*V_V_* [vol.%]
EP-CF-a	1.542 ± 0.007	1.563	0.0	45.6	54.4	0.0	55.8	43.2	0.8 ± 0.2
EP-5PCL-CF-a	1.359 ± 0.005	1.399	6.2	43.2	50.6	8.1	51.5	39.2	1.2 ± 0.1
EP-10PCL-CF-a	1.341 ± 0.005	1.393	10.0	42.0	48.0	12.1	48.7	37.6	1.6 ± 0.1

*w_PCL_* = weight fraction of PCL; *w_EP_* = weight fraction of epoxy resin; *w_CF_* = weight fraction of carbon fibers; *V_PCL_* = volume fraction of PCL; *V_EP_* = volume fraction of epoxy resin; *V_CF_* = volume fraction of carbon fibers; *ρ_exp_* = experimental density of the composites; *ρ_t_* = theoretical density of the composites; *V_V_* = volume fraction of voids.

**Table 5:** Results of TGA tests on the prepared laminates and on the PCL filament.

Sample	T_1%_ [°C]	T_5%_ [°C]	T_dEP_ [°C]	T_dPCL_ [°C]	m_700_ [%]
EP-CF-a	181.1	349.1	377.8	-	72.7
EP-5PCL-CF-a	216.2	344.8	378.5	-	53.4
EP-10PCL-CF-a	262.3	344.8	376.1	-	47.4
PCL filament	316.3	382.1		418.1	0.1

T_1%_ = temperature at 1% of weight loss; T_5%_ = temperature at 5% of weight loss; T_dEP_ = temperature associated to the maximum mass loss rate of epoxy; T_dPCL_ = temperature associated to the maximum mass loss rate of PCL; m_700_ = residual mass at 700°C.

**Table 6:** Results of flexural tests and short beam shear strength tests on the prepared laminates.

	Flexural Modulus [GPa]	Flexural Strength [MPa]	Flexural Strain at Break [%]	P_m_ [N]	ILSS [MPa]
EP-CF	60.8 ± 3.4	621.4 ± 72.3	1.3 ± 0.1	2016 ±139	67.0 ± 3.7
EP-5PCL-CF	66.1 ± 3.4	698.4 ± 37.3	1.3 ± 0.2	4342 ± 155	50.9 ± 1.5
EP-10PCL-CF	65.0 ± 2.7	512.1 ± 34.7	1.1 ± 0.1	5073 ± 146	40.6 ± 1.3

**Table 7:** Electrical resistivity of the prepared laminates.

	Electrical Resistivity [Ω mm]
EP-CF-a	0.065 ± 0.014
EP-5PCL-CF-a	0.052 ± 0.017
EP-10PCL-CF-a	0.051 ± 0.012

**Table 8:** Results of the mode I interlaminar fracture toughness tests on the virgin and healed laminates.

Sample	P_MAX_ [N]		G_I_^NL^ [kJ/m^2^]		Healing Efficiency [%]
	Virgin	Healed	Virgin	Healed	$\eta_{G_{I}}^{NL}$
EP-CF-b	69.7 ± 3.6	10.8 ± 0.8	0.78 ± 0.01	0.01 ± 0.01	0.4 ± 0.2
EP-5PCL-CF-b	123.7 ± 6.5	51.1 ± 9.3	0.97 ± 0.12	0.15 ± 0.04	14.8 ± 4.4
EP-10PCL-CF-b	150.2 ± 15.7	76.4 ± 26.9	0.98 ± 0.05	0.31 ± 0.08	31.5 ± 8.8

**Table 9:** Comparison of healing efficiency values obtained in literature papers on epoxy/PCL matrices (with or without the presence of fibers).

Code	Composite	PCL Content [%wt]	Mending Process	Healing Parameters	Healing Efficiency [%]	Ref.
a	EP/PCL/CF	5.4	30 min at 80 °C	Max load in delamination test	43 ± 7	This work
b	EP/PCL/CF	9.9	30 min at 80 °C	Max load in delamination test	50 ± 7	This work
c	EP/PCL blend	12.5	30 min at 80 °C	Max load in compact tension test	2 ± 1	[22]
d	EP/PCL electrospun	15.0	10 min at 80 °C	Max load in tensile test	45 ± 1	[20]
e	EP/PCL blend	20.0	30 min at 80 °C	Max load in quasi static test	18 ± 5	[50]
f	EP/PCL blend	20.0	30 min at 80 °C	Fracture toughness in impact test	27 ± 6	[50]
g	EP/PCL blend	22.2	30 min at 150 °C	Fracture toughness in tapered double cantilever beam (TDCB) test	70 ± 25	[14]
h	Epoxy/SMP/PCL	23.3	30 min at 84 °C	Max load in single edge notched bending (SENB) test	19 ± 1	[51]
i	EP/PCL blend	25.0	30 min at 80 °C	Max load in compact tension test	11 ± 1	[22]
l	EP/PCL blend	37.5	30 min at 80 °C	Max load in compact tension test	45 ± 4	[22]
m	EP/PCL blend	50.0	30 min at 80 °C	Max load in compact tension test	62 ± 15	[22]

## Data Availability

The data presented in this study are available on request from the corresponding author.
